# Hemodynamic Response Alterations in Sensorimotor Areas as a Function of Barbell Load Levels during Squatting: An fNIRS Study

**DOI:** 10.3389/fnhum.2017.00241

**Published:** 2017-05-15

**Authors:** Rouven Kenville, Tom Maudrich, Daniel Carius, Patrick Ragert

**Affiliations:** ^1^Faculty of Sport Science, Institute for General Kinesiology and Exercise Science, University of LeipzigLeipzig, Germany; ^2^Department of Neurology, Max Planck Institute for Human Cognitive and Brain SciencesLeipzig, Germany

**Keywords:** functional near-infrared spectroscopy (fNIRS), compound movement, motor cortex, hemodynamic response alterations, neuroplasticity

## Abstract

Functional near-infrared spectroscopy (fNIRS) serves as a promising tool to examine hemodynamic response alterations in a sports-scientific context. The present study aimed to investigate how brain activity within the human motor system changes its processing in dependency of different barbell load conditions while executing a barbell squat (BS). Additionally, we used different fNIRS probe configurations to identify and subsequently eliminate potential exercise induced systemic confounders such as increases in extracerebral blood flow. Ten healthy, male participants were enrolled in a crossover design. Participants performed a BS task with random barbell load levels (0% 1RM (1 repetition maximum), 20% 1RM and 40% 1RM for a BS) during fNIRS recordings. Initially, we observed global hemodynamic response alterations within and outside the human motor system. However, short distance channel regression of fNIRS data revealed a focalized hemodynamic response alteration within bilateral superior parietal lobe (SPL) for oxygenated hemoglobin (HbO_2_) and not for deoxygenated hemoglobin (HHb) when comparing different load levels. These findings indicate that the previously observed load/force-brain relationship for simple and isolated movements is also present in complex multi-joint movements such as the BS. Altogether, our results show the feasibility of fNIRS to investigate brain processing in a sports-related context. We suggest for future studies to incorporate short distance channel regression of fNIRS data to reduce the likelihood of false-positive hemodynamic response alterations during complex whole movements.

## Introduction

Recent animal and human studies have provided compelling evidence, that the brain modulates its activity as a function of applied load. In animals, a pioneer study, conducted by Evarts (1968) showed that the brain reorganizes itself when exposed to external force requirements. These results applied to isolated movements and were later supplemented for more complex motion sequences (Georgopoulos et al., 1982).

Similar findings were described in human studies using functional magnetic resonance imaging (fMRI), positron emission tomography (PET) and electroencephalography (EEG). For example, Thickbroom et al. (1998) could demonstrate by means of fMRI that an increase in applied force during a finger flexion task is accompanied by a spread of activation within the contralateral hand area of M1. Further, a much higher activity in contralateral sensory and motor areas of the hand was observed during a “power grip” task (Ehrsson et al., 2000). A positive linear relationship could also be observed between the applied force and the activity in the contralateral primary motor cortex (M1) and primary somatosensory cortex (SSC)(S1) during a hand grip task (Kuhtz-Buschbeck et al., 2008). Accordingly, it could be shown that, for the flexion of forearm and the extension of finger muscles, force and neural activity in the contralateral M1 were proportional to each other, so that the neural activity was amplified with increasing strength (Dai et al., 2001). Additionally, a high correlation was shown for the applied force during the contraction of the index finger and the activity in several areas of the cortex (van Duinen et al., 2008). Further, Ward and Frackowiak (2003) found a high correlation between the height of the blood oxygen level dependent (BOLD) signal (contralateral M1 and S1) and the force applied during a hand grip task. These results were later confirmed by subsequent fMRI studies (Talelli et al., 2008; Ward et al., 2008; Noble et al., 2011). Using PET, Dettmers et al. (1995) observed a logarithmic relationship between power stages during pressing of a Morse key and regional cerebral blood flow changes. Here, M1 showed the strongest correlation between blood flow and applied force. On the other hand, differences in neural activity between a “precision grip” (compression of the thumb and index finger) and a “power grip” (clenching of the fist) were assessed by Takasawa et al. (2003), who found no significant differences in brain activity between “precision grip” and “power grip”.

Similar findings regarding the relation between applied load and associated brain processing were also shown using EEG recordings in humans. For example, during a pinch force task an inverse-linear correlation was seen between the alpha-band activity over the contralateral sensorimotor cortex (SMC) and respective force levels. Exclusively in the gamma-band, increased activities were recorded at the strongest contractions, which was attributed to increased communication of several cortical networks (Mima et al., 1999). Furthermore, recordings of movement related cortical potentials (MRCP) could show that there was a strong association between MRCP amplitudes and respective load/force levels (Siemionow et al., 2000; Slobounov et al., 2002; do Nascimento et al., 2005).

One major drawback of the aforementioned studies is that so far only isolated movements were investigated. Hence, it still remains elusive whether or not a similar load force-brain relationship can also be observed for complex multi-joint whole body movements. Since fMRI and PET are limited to stationary use and EEG is potentially confounded by motion artifacts, such multi-joint whole body movements can obviously not be sufficiently investigated. One potential non-invasive alternative for mobile recordings is functional near-infrared spectroscopy (fNIRS). fNIRS determines site-specific concentration changes of oxygenated (HbO_2_) and deoxygenated (HHb) hemoglobin in analogy to fMRI. Hence, fNIRS is an indirect measure of neural activity by investigating changes in the oxygenation level of the blood (Obrig et al., 1996).

Using fNIRS, it could be shown that brain activity during a finger tapping task is modulated in dependency of the tapping frequency (Obrig et al., 1996). Furthermore, an increased neural activity as a function of grip intensity was described by Bhambhani et al. (2006). Additionally, rises in exerted strength during a handgrip task seem to be accompanied by an increase in neural activity in the ipsilateral premotor cortex (PMC) (Shibuya et al., 2008). Further studies could show that neural activity also increases in primary sensorimotor areas (SM1) and prefrontal cortex (PFC) in response to increased force levels during an unimanual handgrip task (Derosière et al., 2014). Moreover, neural activity in M1 seems to be correlated with the respective force applied during a hand grip task (Derosiere and Perrey, 2012). Finally, significant differences in the activation level of the contralateral M1 were observed in dependency of applied force during unilateral finger flexion (Shibuya et al., 2014).

In accordance with previous non-invasive brain imaging studies, fNIRS has been exclusively used to investigate simple and isolated movements regarding the load/force brain relationship. However, some fNIRS feasibility studies provided compelling evidence that this imaging technique is capable of providing results in complex movement patterns such as walking (Suzuki et al., 2004) or cycling (Shibuya et al., 2004). Therefore, its application is potentially appropriate concerning our fundamental question dealing with investigating the load/force brain relationship in complex whole body movements.

However, previous studies indicated that fNIRS brain recordings especially during complex movement patterns might be confounded by certain systemic variables such as the cardiac output volume (Ide et al., 1998; Giller et al., 2000; van Lieshout et al., 2001) and blood pressure (Harper, 1966; Edwards et al., 2002). Both parameters seem to have a considerable influence on the cerebral blood flow rate, which in turn is closely related to the measured fNIRS signal (Madsen et al., 1995; Smielewski et al., 1995). Alterations in blood pressure and/or cardiac output volume during task performance might influence and therefore confound the fNIRS signal. Additionally, two distinct vascular supply systems, the cerebral vascular and the subcutaneous vascular system of the scalp are located within the penetration range of the near infrared light. The transmitted light can thus detect chromophore concentrations of both systems (Tachtsidis and Scholkmann, 2016), which is potentially unfavorable. Studies showed that chromophore progressions differ between both systems, when monitoring their behavior during exercise (Auger et al., 2016). However, recent methodological approaches have been developed to reduce such potential confounds during fNIRS recordings. For example, it has been proposed that reducing the penetration depth of the near-infrared light might potentially be effective in differentiating between fNIRS signals that are of cerebral and/or extracerebral origin. In fact, Yücel et al. (2015) demonstrated the effectiveness of a short-separation regression in that context.

Based on the aforementioned findings and limitations with respect to fNIRS, the present study aimed to investigate how brain activity within the human motor system changes its processing in dependency of different load conditions while executing a barbell squat (BS). Furthermore, we investigated different fNIRS configurations to expose global effects and applied a short distance channel regression to eliminate potential confounders. More specifically, we hypothesized, in accordance to previous findings using simple and isolated movements of the upper limbs, that neural processing in the human motor system reorganizes as a function of the applied load during a complex whole body movement. Additionally, we expected a more localized brain activity in movement-related brain regions such as M1 area when fNIRS recordings were paired with short distance channel regression of the fNIRS signal.

## Materials and Methods

### Participants

A total number of 10 healthy, male participants (age: 25.7 ± 2.2 years (mean ± SD)) were enrolled in the present study. The study was approved by the local ethics committee of the Medical Faculty at the University of Leipzig (ref.-nr. 410-15-16112015) and all participants gave their written informed consent to participate in the experiments in accordance with the Declaration of Helsinki. Prior to participation, each participant was instructed, in terms of content and about the procedure. Participants were excluded from the present study in case the following exclusion criteria were present: neurological/ psychological disease, intake of central acting drugs, caffeine or alcohol intake 24 h before the experiment, acute, chronic and/or inadequately regenerated pathologies of the knee joint, the ankle joints and/or the spine to minimize the risk of injury. The experiment consisted of a randomized cross-over design where each participant underwent three experimental sessions, separated by at least 3 days. The only difference between sessions was a different fNIRS channel arrangement that was used to capture different brain regions during task performance (for details see below). All participants were familiar with and/or experienced in the task (BS).

### Behavioral Task (Barbell Squat)

At the beginning of each experimental session, a dynamic maximum strength test was conducted using the BS with additional load (one repetition maximum (1RM)). For that purpose, participants had to perform the BS with a stepwise increase of additional loads. After each successful BS, the load was increased individually until participants could complete no more than three correct BS movements. The respective absolute maximum force value, i.e., the load with which the subject can exactly complete a repetition (rep) (kg), was calculated from this value. The calculation was made using the following “Epley equation”: 1RM = Load × (1 + (0.033 × rep)) (Epley, 1985).

**Figure 1:**
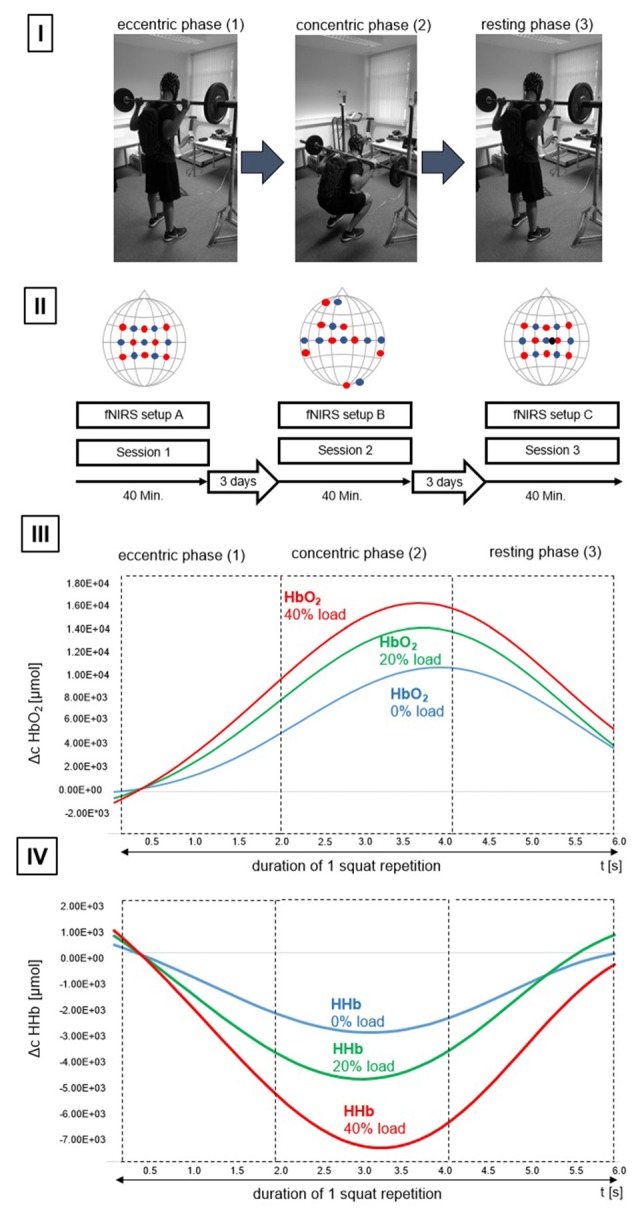
**Study design. (I)** Subdivision of the activation phase (barbell squat (BS)). (1) Initiation of the eccentric phase, (2) end of the eccentric phase and subsequent initiation of the concentric phase, (3) end of the concentric phase and concurrently the initial position of the resting phase (R). **(II)** Overview of the configurations used per session. Illustrated are configurations (functional near-infrared spectroscopy (fNIRS) setup (A), (B) and (C)). Detectors are shown as blue dots, transmitters are shown as red dots. The short—distance detector is shown as a black dot. Also illustrated are time of one session (40 min), as well as the time between individual sessions (3 days). **(III)** Progression of oxygenated hemoglobin (HbO_2_) for one repetition. Data is based on mean average progression across all participants. Chromophore progression is illustrated for L0% (blue line), L20% (green line) and L40% (red line) and divided to showcase the different movement phases. **(IV)** Progression of deoxygenated Hemoglobin (HHb) for one repetition. Data is based on mean average progression across all participants. Chromophore progression is illustrated for L0% (blue line), L20% (green line) and L40% (red line) and divided to showcase the different movement phases. Please note that the participant displayed in (I) gave written informed consent to use the pictures illustrating the study design.

Subsequently, participants had to perform the BS under three different experimental conditions (L0%, L20%, L40%) representing the execution of BS with 0% of 1RM (L0%), BS with 20% 1RM (L20%) and BS with 40% 1RM (L40%). The order of the conditions was randomized across participants. During each condition a total number of five BS was performed within one activity block. The movement during BS was subdivided into three chronologically standardized phases: (A) eccentric, (B) concentric phase and (C) repetition pause (see Figure 1). The duration of each phase was 2 s giving a total block length of 30 s. A total number of five blocks per condition was performed resulting in 15 blocks per session. Between each block there was a rest period of 30 s which lead to a total recording time of 15 min per session. The total duration of each session was approximately 25 min.

To ensure a standardized movement amplitude of 95° in the knee joint during BS, a gyroscope was attached to the distal quarter of the vastus lateralis muscle of each subject and provided an acoustic feedback as soon as the desired angle was reached. The default position during BS was an external rotation of the shoulder joint (articulatio humeri), which was paired with flexed elbows and slightly adducted shoulder blades. In this position, the hands were at the level of the larynx, 7–8 cm to the left or to the right of the head, respectively, so that the dumbbell bar rested on the trapezius muscle and the open palms. To ensure a timely accurate BS, the respective phase was displayed on a PC screen.

### Neuronavigation

To transform topographic maps from the recorded hemodynamic responses, the fNIRS probe positions (x, y, z space) were registered to Montreal Neurological Institute (MNI) space. This was done via the neuronavigation device: Brainsight™ (Version 2, Rogue Research, Montreal, QC, Canada). Subsequently, the respective MNI coordinates for each fNIRS probe were fed into a probabilistic atlas to allow the assignment of the hemodynamic response alterations during BS with different loads to a specific brain region. For that purpose, the Juelich histological (cyto- and myelo-architectonic) atlas was used within the program FSL (FMRIB Software Library v5.0, Created by the Analysis Group, FMRIB, Oxford, UK; for reference see e.g., Eickhoff et al., 2005, 2006, 2007).

### NIRS—Recording and Analysis

Hemodynamic response alterations were assessed using the portable fNIRS system NIRSport™ (NIRx Medical Technologies, New York, NY, USA). For that purpose, different fNIRS cap/probe arrangements were utilized comprising of an 8 “source” (transmitter) × 8 “receiver” (detector) probe arrangement. The center of the cap was placed according to the international 10-20 system over the vertex (Cz) of each participant. Cz was determined over the intersection of the courses: nasion-inion and left preauricular point-right preauricular point according to Jurcak et al. (2007). In general, three distinct fNIRS cap configurations, one for each session, were used in the present study. The aim of using three distinct cap configurations was motivated by the fact that we intended to disentangle hemodynamic response alterations as a function of the applied load, (A) specifically within the bilateral sensorimotor system, including cortical motor regions such as primary motor cortex (M1), PMC, supplementary motor area (SMA), inferior parietal lobe (IPL) and superior parietal lobe (SPL) and (B) in brain areas that are not predominantly task related (such as auditory, frontal and visual areas, see Figure 1(II)). Respective brain regions were targeted by a 10-20 system transfer method, introduced by Lancaster et al. (2000).

Additionally, we used another fNIRS cap configuration (C) to eliminate potential fNIRS confounders, such as extracerebral blood flow alterations. For that purpose, we used an additional short-distance probe within configuration (A) with an inter-optode distance of 1 cm, as opposed to the inter-optode distance for all other channels of our configurations (3 cm). For a detailed overview of the respective fNIRS probe setups please see Figure 1(II). The order of the sessions was counterbalanced across participants.

During fNIRS recordings, we used a sampling rate of 7.81 Hz. Spectroscopically, the NIRSport™ operated via a so-called “continuous wave” method. This means that the sources emit light at constant frequency and intensity (Scholkmann et al., 2014).

After each session, hemodynamic response alterations were analyzed (post-processing steps) using an NIRS-SPM (statistical parametric mapping) tool (Ye et al., 2009), which was included in the analysis software nirsLAB (v2014, NIRx Medical Technologies, New York, NY, USA). Initially, the length of task (BS) onsets and rest periods (R, no BS) were defined (each block had trial length of 30 s). Signal-to-noise performance of individual channels was evaluated via variation coefficients (CV) which is a common method for multi-channel fNIRS measurements (Schmitz et al., 2005; Schneider et al., 2011; Piper et al., 2014). A CV is mathematically defined as 100 times the standard deviation divided by the mean value, where the standard deviation and mean are computed from all the raw-data values in the measurement time series. fNIRS channels were removed when respective channels exceeded a variation coefficient of 15% (Piper et al., 2014). Subsequently data was subjected to a baseline correction (10 s before onset) and then filtered (band pass filter: low cutoff frequency = 0.01 Hz high cutoff frequency = 0.2 Hz) to attenuate high-frequency noise and cardiovascular artifacts (Huppert et al., 2009).

Conditions (BS during 0% 1RM (no load, L0%), 20% 1RM (L20%) and 40% 1RM (L40%) were modeled using a “boxcar” function folded with the canonic hemodynamic response function according to Helmich et al. (2013). Data were then transformed into a General Linear Model (GLM) estimation to obtain beta values, which were indicators of the correlation between the obtained signal and the model-immanent regressors, the conditions and the timely progression of the distinct chromophores HbO_2_ and HHb.

Apart from the aforementioned post-processing steps for fNIRS cap configurations (A) and (B), a fourth condition, corresponding to the averaged chromophore progression over the individual short-distance channels, was included in the GLM for the session which included the short distance channel (fNIRS configuration (C)). In general, post-processing steps were comparable across fNIRS configurations except that for (C), the individually averaged short distance channel profile was extracted for each participant and subsequently integrated into the GLM as a regressor. On a group level, these individual models were grouped together and used for final group statistics.

Data were subsequently analyzed using the software package SPSS Statistics 22 (IBM, Armonk, NY, USA). Initially, normal distribution of data was evaluated by Shapirro-Wilk-Test. All data were normally distributed. Hence, only parametric tests were used for subsequent statistical analyses. To assess statistical differences in hemodynamic response alterations among the 0%, 20% and 40% 1RM barbell load levels, a repeated measures ANOVA was used to test for significant effects of force level (0%, 20% and 40% barbell load) on beta-values of HbO_2_ and HHb. If necessary, data were corrected for sphericity using Greenhouse-Geisser correction. Partial eta-squared ($\eta_{\text{p}}^{\text{2}}$) for ANOVA’s are provided as measures of effect size and used to aid in the interpretation of inferential statistics.

When appropriate, a *post hoc* analysis with paired *t*-tests was used to detect significant differences between load levels for each channel. Furthermore, paired *t*-tests were performed for comparing load levels and baseline values (BL) in respective channels for each group. Bonferroni corrected *p*-values (configuration (A) *p* < 0.0023; configuration (B) *p* < 0.0038; configuration (C) *p* < 0.0023) were used as a threshold for significance. Visualization of hemodynamic response alterations was performed using the Brain Function Mapping Tool from Wang et al. (2016).

## Results

The mean 1RM value during BS was 98 kg (±11.35 SD) and did not differ between sessions since 1RM values were identical within participants.

### Hemodynamic Response Alterations within Sensorimotor Areas (fNIRS Configuration (A))

Repeated measures ANOVA revealed significant changes in HbO_2_ and HHb beta-values between all load levels for HbO_2_ (*F*_(1.55,341.10)_ = 528.55, *p* < 0.0001, $\eta_{\text{p}}^{\text{2}}$ = 0.71) and HHb (*F*_(1.47,322.01)_ = 107.63, *p* < 0.0001, $\eta_{\text{p}}^{\text{2}}$ = 0.33).

**Figure 2:**
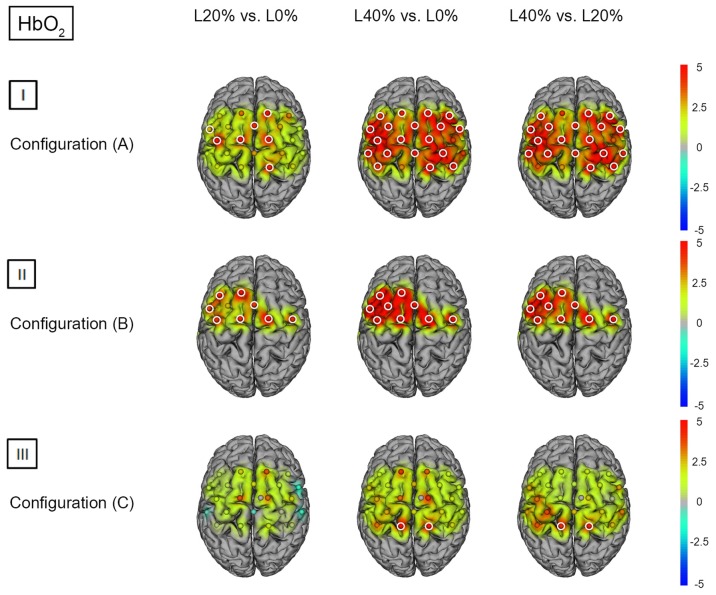
**HbO_2_*t* statistics for fNIRS configurations (A, B) and (C)**. Channels (centered between transmitters and detectors) are shown for each image. Decreases in HbO_2_ responses are illustrated in dark blue, increases in HbO_2_ responses are illustrated in dark red. The entire range is illustrated in the attached legend. Significant channels are marked with white circles. **(I)** Areas that are significantly more active for BS at L20% vs. L0%, L40% vs. L0% and L40% vs. L20% for configuration (A). All images are thresholded at *p* < 0.0023. **(II)** Areas that are significantly more active for BS at L20% vs. L0%, L40% vs. L0% and L40% vs. L20% for configuration (B). All images are thresholded at *p* < 0.0038. **(III)** Areas that are significantly more active for BS L20% vs. L0%, L40% vs. L0% and L40% vs. L20% for configuration (C). All images are thresholded at *p* < 0.0023.

**Figure 3:**
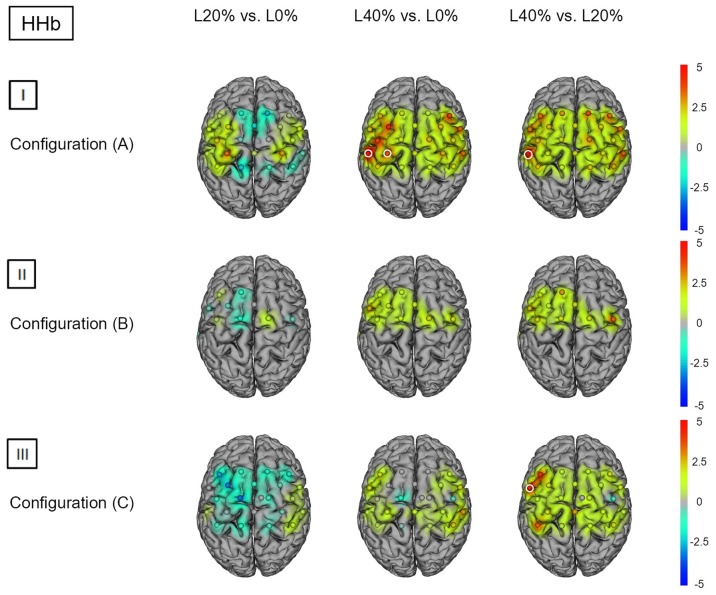
**HHb *t* statistics for fNIRS configurations (A, B) and (C)**. Channels (centered between transmitters and detectors) are shown for each image. Decreases in HbO_2_ responses are illustrated in dark blue, increases in HbO_2_ responses are illustrated in dark red. The entire range is illustrated in the attached legend. Significant channels are marked with white circles. **(I)** Areas that are significantly more active for BS at L20% vs. L0%, L40% vs. L0% and L40% vs. L20% for configuration (A). All images are thresholded at *p* < 0.0023. **(II)** Areas that are significantly more active for BS at L20% vs. L0%, L40% vs. L0% and L40% vs. L20% for configuration (B). All images are thresholded at *p* < 0.0038. **(III)** Areas that are significantly more active for BS L20% vs. L0%, L40% vs. L0% and L40% vs. L20% for configuration (C). All images are thresholded at *p* < 0.0023.

*Post hoc* analysis revealed significant hemodynamic response alterations for L20% vs. L0% in M1, right PMC, SMA, left IPL and right SPL for HbO_2_. No such effects could be observed for HHb. Comparing L40% vs. L0% revealed significant hemodynamic response alterations in all channels except channels 12, 18 and 22 for HbO_2_. For HHb, significant hemodynamic response alterations could be shown in left SSC and IPL when comparing L40% vs. L0%. Comparisons between L40% vs. L20% revealed significant hemodynamic response alterations in all channels except channels 4, 9, 12 and 18 for HbO_2_, while the comparison for HHb revealed no statistical differences (see Figures 2(I) and 3(I); for detailed statistics please see also Supplementary Table S1).

**Figure 4:**
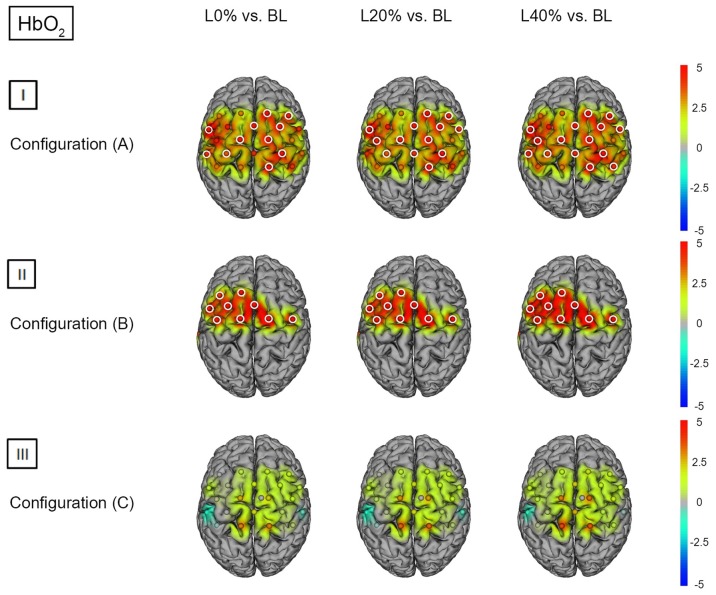
**HbO_2_*t* statistics for fNIRS configurations (A, B) and (C)**. Channels (centered between transmitters and detectors) are shown for each image. Decreases in HbO_2_ responses are illustrated in dark blue, increases in HbO_2_ responses are illustrated in dark red. The entire range is illustrated in the attached legend. Significant channels are marked with white circles. **(I)** Areas that are significantly more active for BS at L0% vs. baseline values (BL), L20% vs. BL and L40% vs. BL for configuration (A). All images are thresholded at *p* < 0.0023. **(II)** Areas that are significantly more active for BS at L0% vs. BL, L20% vs. BL and L40% vs. BL for configuration (B). All images are thresholded at *p* < 0.0038. **(III)** Areas that are significantly more active for BS at L0% vs. BL, L20% vs. BL and L40% vs. BL for configuration (C). All images are thresholded at *p* < 0.0023.

**Figure 5:**
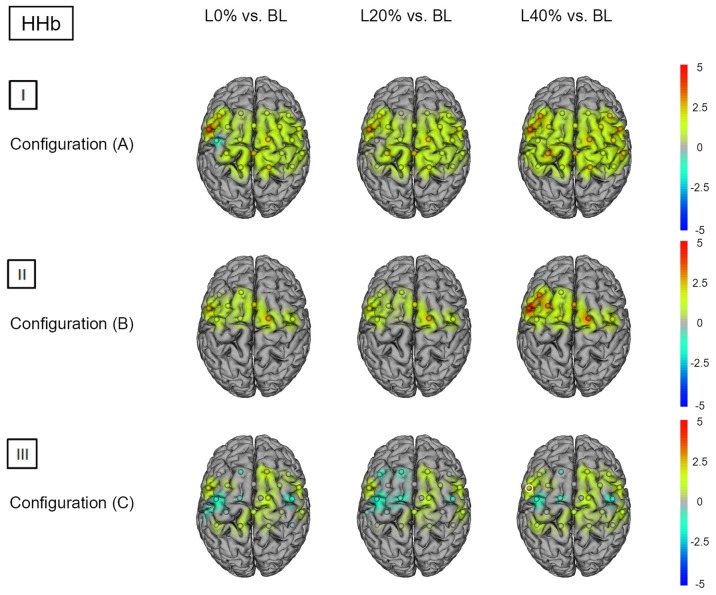
**HHb *t* statistics for fNIRS configurations (A, B) and (C)**. Channels (centered between transmitters and detectors) are shown for each image. Decreases in HbO_2_ responses are illustrated in dark blue, increases in HbO_2_ responses are illustrated in dark red. The entire range is illustrated in the attached legend. Significant channels are marked with white circles. **(I)** Areas that are significantly more active for BS at L0% vs. BL, L20% vs. BL and L40% vs. BL for configuration (A). All images are thresholded at *p* < 0.0023. **(II)** Areas that are significantly more active for BS at L0% vs. BL, L20% vs. BL and L40% vs. BL for configuration (B). All images are thresholded at *p* < 0.0038. **(III)** Areas that are significantly more active for BS at L0% vs. BL, L20% vs. BL and L40% vs. BL for configuration (C). All images are thresholded at *p* < 0.0023.

Comparing BS during L0% vs. BL revealed significant hemodynamic response alterations for HbO_2_ in bilateral M1, SSC, SMA, SPL, left IPL and right PMC. When comparing BS during L20% and L40% vs. BL, a similar effect was observed for HbO_2_. For HHb we did not find significant differences (see Figures 4(I) and 5(I); for detailed statistics please see also Supplementary Table S4).

### Hemodynamic Response Alterations Outside of Sensorimotor Areas (fNIRS Configuration (B))

Repeated measures ANOVA revealed significant changes in HbO_2_ and HHb beta-values between all load levels for HbO_2_ (*F*_(1.36,176.01)_ = 208.54, *p* < 0.0001, $\eta_{\text{p}}^{\text{2}}$ = 0.62) and HHb (*F*_(1.69,217.56)_ = 14.64, *p* < 0.0001, $\eta_{\text{p}}^{\text{2}}$ = 0.10).

*Post hoc* analysis revealed significant hemodynamic response alterations for L20% vs. L0% in bilateral M1, IPL, SMA, and PMC for HbO_2_, while no such effect could be observed for HHb. Comparing L40% vs. L0% revealed comparable significant hemodynamic response alterations in all channels except channels 1, 12 and 13 for HbO_2_. For HHb, L40% vs. L0% showed no significant hemodynamic response alterations. Comparisons between L40% vs. L20% revealed significant hemodynamic response alterations in all channels except channels 1 and 13 for HbO_2_. The comparison for HHb revealed no significant effects (see Figures 2(II) and 3(II); for detailed statistics please see also Supplementary Table S2).

Comparing BS during L0%, L20% and L40% vs. BL revealed significant hemodynamic response alterations for HbO_2_ in all fNIRS channels except channels 1 and 13. No significant modulation could be observed for HHb (see Figures 4(II) and 5(II); for detailed statistics please see also Supplementary Table S5).

### Hemodynamic Response Alterations within Sensorimotor Areas after Short Distance Channel Regression (fNIRS Configuration (C))

Repeated measures ANOVA revealed significant changes in HbO_2_ and HHb beta-values between all load levels for HbO_2_ (*F*_(1.74,398.62)_ = 102.52, *p* < 0.0001, $\eta_{\text{p}}^{\text{2}}$ = 0.31) and HHb (*F*_(2.00,452.98)_ = 19.59, *p* < 0.0001, $\eta_{\text{p}}^{\text{2}}$ = 0.08).

*Post hoc* analysis between barbell load levels during BS revealed no significant hemodynamic response alterations for L20% vs. L0% for either chromophore. Comparing L40% vs. L0% revealed significant hemodynamic response alterations in bilateral SPL for HbO_2_, while no significant effects could be observed for HHb. For HbO_2_, L40% vs. L20% also revealed significant hemodynamic response alterations in bilateral SPL. The same comparison for HHb revealed significant effects in left PMC (see Figures 2(III) and 3(III); for detailed statistics please see also Supplementary Table S3).

Comparing BS during L0%, L20% and L40% vs. BL showed no significant hemodynamic response alterations for HbO_2_. For HHb, only L40% vs. BL revealed a significant hemodynamic response alterations in left PMC (see Figures 4(III) and 5(III); for detailed statistics please see also Supplementary Table S6).

## Discussion

Based on our results, we provide novel evidence that fNIRS is capable of assessing hemodynamic response alterations within the human motor system during the execution of a complex whole body movement (BS). Furthermore, we could demonstrate that a short distance channel regression of fNIRS data seems to be suitable of improving fNIRS data analysis. Here, short distance channel regression revealed a focalized hemodynamic response alteration within bilateral SPL during BS performed at 40% 1RM. Hence, the present study is the first of its kind to reveal brain regions that are potentially involved in the execution of a BS, as well as the first study to report correspondence between cortical activity and force requirements in a complex whole body movement. This indicates that sensorimotor areas, such as bilateral SPL, modulate their activity as a function of applied load during the execution of a complex whole body movement (BS). Further, our results are in accordance with previous findings, who also reported force-dependent modulation of cortical activity during isolated simple movements (Bhambhani et al., 2006; Rasmussen et al., 2007; Shibuya et al., 2008, 2014; Rupp and Perrey, 2009; Shibusawa et al., 2009; Derosiere and Perrey, 2012). However, we did not see significant hemodynamic response alterations in M1 when comparing different barbell load levels which contrasts with previous studies (Shibuya et al., 2008, 2014; Shibusawa et al., 2009; Derosiere and Perrey, 2012; Derosière et al., 2014). Even though we can only speculate about these divergent findings, it seems reasonable to assume that especially during complex whole body movements extra-cortical brain regions such as the cerebellum might play a pivotal role. In fact, several brain imaging studies could demonstrate that apart from cortical brain regions such as M1 (Shibuya et al., 2008), the cerebellum also shows a similar load-related brain reorganization (Dai et al., 2001; Sehm et al., 2010; Noble et al., 2011; Spraker et al., 2012; Charles et al., 2013). Furthermore, previous fMRI studies could show that cerebellar-parietal circuits modulate movement complexity in unilateral sequential finger movements, underlining the importance of subcortical output paths in complex movements (Haslinger et al., 2002).

Obviously, fNIRS is not capable of assessing extra- and/or sub-cortical regions because of its limited penetration depth. Therefore, other approaches must be adopted to shed more light on the role of the cerebellum during force-dependent alterations in complex whole body movements.

It is also important to note that hemodynamic response alterations during the BS were primarily observed for HbO_2_. To date, it has been controversially discussed which chromophore (HbO_2_ or HHb) is more reliable and valid in respect to the indirect measurement of cortical activity via fNIRS (Schroeter et al., 2002; Strangman et al., 2002; Obrig and Villringer, 2003). During a test-retest reliability study for event-related fNIRS measurements, Plichta et al. (2006) concluded that, though locally more specific, HHb is limited regarding reproducibility of the localization over time and over subjects, when compared to HbO_2_. In accordance, several fNIRS studies describe arbitrary behavior of HHb (Maki et al., 1996; Watanabe et al., 1996; Miyai et al., 2001). For example, Miyai et al. (2001) observed both increases and decreases in concentration of HHb for a standardized task between participants. Contrarily, several studies could draw conclusions on active areas based on HHb courses (Durduran et al., 2004; Sato et al., 2007; Shibuya et al., 2008). Regarding the assessment of neural activity with fNIRS, there are studies which use a simultaneous increase in HbO_2_ and decrease in HHb as indicators of neural activity (Shibuya et al., 2014). Also, there are fNIRS studies dealing with force/load-activity relationships in which only the increase of HbO_2_ and a constant behavior of HHb were used as indicators of neural activation (Derosiere and Perrey, 2012). Lastly, to our knowledge, no fNIRS studies investigating motor tasks have established neural activity by specific HHb courses combined with constant HbO_2_ behavior, underlining the fact, that merely using HHb behavior to assess neural activity in fNIRS measurements, seems to be too unreliable.

Our results show broad regional variations regarding significant hemodynamic response alterations for HHb. The absence of a regionally consistent HHb response can generally be attributed to its vascular as well as its hemodynamic dependencies. Thus, HHb behavior varies depending on the degree of oxygenation in venous blood (Hoshi et al., 2001; Miyai et al., 2001). In addition, the individuality of the diameters of distinct arteries or venules can also influence the task-dependent HHb behavior (Hoshi et al., 2001). Additionally, a physiological crosstalk between the chromophores (HbO_2_ and HHb) can potentially be induced depending on the chosen wavelengths. Although many studies consider a value of 830 nm as the best maximum value (Yamashita et al., 2001; Strangman et al., 2003; Sato et al., 2004; Okui and Okada, 2005; Kawaguchi et al., 2008) there are also studies that show contrary results (Uludag et al., 2004; Correia et al., 2010). Thus, there is potentially the risk of negating certain physiological chromophore responses due to improper selection of wavelength combinations. However, since the wavelengths in the present study (760 nm and 850 nm) correspond to the optimal ranges postulated in current literature, this risk can be considered as minor. Lastly, it can be speculated that although we standardized the task (BS) as much as possible, a differing peripheral activation pattern during the execution of BS between individuals cannot be ruled out. Accordingly, a comprehensive review by Clark et al. (2012) highlighted several determinants which might cause a change in peripheral activation during a BS, *inter alia* “depth of the BS”, “width of the stance”, “load” and “torso power”. Consequently, it could be the case that the varying peripheral activity patterns provoke differential cortical HHb responses between individuals, which vary in such a way that there is no regional uniform HHb response in the group analysis and thus no HHb hemodynamic response alteration. Finally, it is also possible that eccentric and concentric phases differ regarding their underlying cortical initiation and control during performance of BS, which could potentially result in spatially heterogenic hemodynamic response alterations across the fNIRS channels. It could be shown that movement-related cortical potentials (MRCP’s; Fang et al., 2001, 2004) as well as BOLD signals (Yao et al., 2014) vary between eccentric and concentric contractions. No studies have been investigating this phenomenon in complex whole-body movements with fNIRS so far, which is why we can’t draw definite conclusions with regards to this issue. It should be mentioned that the transfer from eccentric to concentric phases is more distinct and pronounced in isolated movements when compared to BS (for a detailed view of the respective chromophore progressions for concentric and eccentric phases, please see Figures 1(III,IV)). This effect should be investigated in complex whole body movements in the future.

As mentioned above, our results show global hemodynamic response alterations within and outside the sensorimotor system during BS without short-distance channel regression. These results indicate that the obtained signal might be confounded by several potential factors such as certain systemic variables like cardiac output volume (Ide et al., 1998; Giller et al., 2000; van Lieshout et al., 2001) and blood pressure (Harper, 1966; Edwards et al., 2002). Both have a considerable influence on the cerebral blood flow rate, which in turn is closely related to the measured fNIRS signal (Madsen et al., 1995; Smielewski et al., 1995). Blood pressure, in most cases the MAP (mean arterial pressure), as well as cardiac output volume, are subject to potential modulators of various types. Daily movements, for example diffraction in the shoulder joint (Minati et al., 2011), but also visual stimulations (Minati et al., 2009), i.e., influences free from movement, manipulate the blood pressure. Cardiac output is strongly modulated by its close association with the acute state of the vegetative nervous system (VNS), above all through sporting activity (Astrand et al., 1964). It is therefore important to observe systemic influences and, at best, to survey them in parallel to be able to make conclusions about the causes of the derived signal with maximum precision. However, to date, there is no solid methodological approach to continuously monitor blood pressure during physical activity.

Additionally, extracerebral blood flow of the scalp can also confound the fNIRS signal. Two distinct vascular supply systems, the cerebral vascular system and the subcutaneous vascular system of the scalp are located within the penetration range of near-infrared light. The transmitted light can thus detect chromophore concentrations stemming from both vascular systems (Tachtsidis and Scholkmann, 2016).

As a first step to control for this potential confounder we incorporated a short distance channel in our fNIRS configuration (C). Subsequently this channel was utilized as a regressor to potentially eliminate the influence of extracerebral hemodynamics on the fNIRS signal. In recent literature, multi-channel configurations, i.e., configurations that incorporate multiple short and long distance channels, have been discussed and used to potentially eliminate extracerebral signal components (Gagnon et al., 2011, 2012; Zhang et al., 2015). It is currently regarded as the most promising approach to globally correct the fNIRS signal. In the present study, the argument can be made, that a single short distance channel is insufficient to detect and globally adjust for extracerebral confounders. However, several studies could show that altering systemic influences, for example cardiac pulsations, are globally homogenous across the scalp (Tian et al., 2011; Zhang et al., 2015), although there are reports which argue the converse (Gagnon et al., 2012). From our point of view, this validates the usage of one short distance channel. Still, it seems worthwhile to shift future fNIRS measurements, especially those investigating motor tasks, toward multi-distance measurements of the whole brain.

Finally, it should be mentioned that the validity of fNIRS during complex movements should be addressed in more detail by future studies. For example, Suzuki et al. (2004) did not observe walking-speed dependent modulations in sensorimotor areas, while during cycling, Auger et al. (2016) in fact reported power-dependent fNIRS alterations. These divergent findings, in light with our findings, point to task-dependent fNIRS modulations and therefore give no definite answer regarding the validity of fNIRS especially during complex movements.

## Conclusion

In the present study, we provide novel evidence that performing the BS leads to hemodynamic response alterations within the human motor system after short-distance channel regression of the fNIRS signal. Furthermore, hemodynamic responses vary as a function of the applied load during the complex whole body movement *BS* as previously shown for simple and isolated movements (Shibuya et al., 2014). Future studies should further investigate: (1) the potential role of systemic factors; and (2) the validity of fNIRS during complex whole body movements. This seems to be of pivotal interest in studies aiming to assess neural activation during the execution of complex movements in the context of sport.

## Author Contributions

RK and PR designed the experiment. RK performed and analyzed the study. TM and DC helped in data acquisition and analysis. RK and PR wrote the article. All authors interpreted and discussed the results.

## Conflict of Interest Statement

The authors declare that the research was conducted in the absence of any commercial or financial relationships that could be construed as a potential conflict of interest.
