# Supplementary material for: Hemodynamic Response Alterations in Sensorimotor Areas as a Function of Barbell Load Levels during Squatting: An fNIRS Study
Source: Front Hum Neurosci. 2017 May 15;11:241. doi: 10.3389/fnhum.2017.00241 (PMC5430058; doi:10.3389/fnhum.2017.00241)
Supplement: Supplementary file 1 [file Table_1_v1.pdf]

**Table 1.** Results of post – hoc paired t – tests between barbell load levels for configuration (A).

| | Post – hoc tests | | | | | | | | | | | |
|---|---|---|---|---|---|---|---|---|---|---|---|---|
| | $HbO_2$ | | | | | | HHb | | | | | |
| | 20% versus 0% | | 40% versus 0% | | 40% versus 20% | | 20% versus 0% | | 40% versus 0% | | 40% versus 20% | |
| Channel | *t* | *p* | *t* | *p* | *t* | *p* | *t* | *p* | *t* | *p* | *t* | *p* |
| 1 | 2.671 | .0256 | 8.439 | <.0001* | 5.710 | .0003* | 1.619 | .1399 | 2.383 | .0410 | 3.212 | .0106 |
| 2 | 2.204 | .0550 | 4.322 | .0019* | 4.570 | .0013* | .649 | .5323 | 2.628 | .0274 | 3.284 | .0095 |
| 3 | 1.607 | .1426 | 5.340 | .0005* | 6.172 | .0002* | .887 | .3980 | 6.480 | .0001* | 4.980 | .0008* |
| 4 | 2.080 | .0673 | 4.265 | .0021* | 3.730 | .0047 | 1.803 | .1048 | 2.324 | .0452 | 2.463 | .0360 |
| 5 | 4.942 | .0008* | 11.145 | <.0001* | 9.446 | <.0001* | 2.322 | .0454 | 3.761 | .0045 | 2.501 | .0338 |
| 6 | 2.094 | .0657 | 4.748 | .0010* | 5.167 | .0006* | 2.272 | .0492 | 4.344 | .0019* | 2.513 | .0332 |
| 7 | 1.927 | .0861 | 4.923 | .0008* | 5.291 | .0005* | 3.275 | .0096 | 3.712 | .0048 | 1.836 | .0995 |
| 8 | 4.885 | .0009* | 6.366 | .0001* | 6.058 | .0002* | -.359 | .7279 | 1.972 | .0800 | 2.144 | .0607 |
| 9 | 3.357 | .0084 | 4.537 | .0014* | 4.091 | .0027 | -1.517 | .1636 | 2.014 | .0749 | 2.785 | .0212 |
| 10 | 4.212 | .0023* | 6.613 | .0001* | 5.013 | .0007* | -2.095 | .0657 | 1.692 | .1249 | 2.147 | .0603 |
| 11 | 4.903 | .0008* | 7.748 | <.0001* | 7.574 | <.0001* | -2.353 | .0431 | 1.151 | .2795 | 3.599 | .0058 |
| 12 | 2.122 | .0628 | 2.948 | .0163 | 2.833 | .0196 | -2.245 | .0514 | 1.246 | .2443 | 1.557 | .1539 |
| 13 | 2.936 | .0166 | 5.241 | .0005* | 4.674 | .0012* | .577 | .5782 | 2.006 | .0758 | 2.236 | .0522 |
| 14 | 4.666 | .0012* | 6.728 | .0001* | 6.166 | .0002* | -.747 | .4740 | 1.585 | .1475 | 2.354 | .0431 |
| 15 | 4.795 | .0010* | 6.085 | .0002* | 5.145 | .0006* | -.075 | .9422 | 2.493 | .0342 | 3.081 | .0131 |
| 16 | 2.676 | .0254 | 6.558 | .0001* | 5.542 | .0004* | .535 | .6054 | 2.546 | .0314 | 2.382 | .0411 |
| 17 | 2.701 | .0244 | 8.627 | <.0001* | 10.742 | <.0001* | 2.936 | .0166 | 2.627 | .0275 | 2.220 | .0536 |
| 18 | 1.508 | .1658 | 3.947 | .0034 | 3.555 | .0062 | 1.051 | .3205 | 2.267 | .0496 | 2.084 | .0668 |
| 19 | 3.187 | .0111 | 7.680 | <.0001* | 6.747 | .0001* | .319 | .7571 | 3.768 | .0044 | 3.275 | .0096 |
| 20 | 1.206 | .2585 | 5.914 | .0002* | 7.020 | .0001* | 1.949 | .0831 | 3.475 | .0070 | 3.353 | .0085 |
| 21 | 2.174 | .0578 | 4.968 | .0008* | 5.255 | .0005* | -.893 | .3952 | 2.065 | .0689 | 2.767 | .0219 |
| 22 | 1.412 | .1915 | 3.915 | .0035 | 4.526 | .0014* | -.449 | .6614 | 3.270 | .0097 | 3.496 | .0068 |

*Note*: Asterisks indicate significant effects between barbell load levels, after correction for multiple comparisons (adjusted p – value $p < 0.0023$). Please note that data listed below (t – statistics), corresponds to the topographic images in Figures 2 (I) and 3 (I).

**Table 2.** Results of post – hoc paired t – tests between barbell load levels for configuration (B).

| | Post – hoc tests | | | | | | | | | | | |
|---|---|---|---|---|---|---|---|---|---|---|---|---|
| | $HbO_2$ | | | | | | HHb | | | | | |
| | 20% versus 0% | | 40% versus 0% | | 40% versus 20% | | 20% versus 0% | | 40% versus 0% | | 40% versus 20% | |
| Channel | *t* | *p* | *t* | *p* | *t* | *p* | *t* | *p* | *t* | *p* | *t* | *p* |
| 1 | 1.101 | .2995 | 2.302 | .0468 | 2.160 | .0591 | -1.624 | .1388 | -2.207 | .0547 | -.771 | .4607 |
| 2 | 5.969 | .0002* | 9.328 | <.0001* | 6.929 | <.0001* | 1.348 | .2106 | 2.280 | .0486 | 2.083 | .0669 |
| 3 | 4.277 | .0021* | 6.180 | .0002* | 5.462 | .0004* | -.334 | .7462 | 2.121 | .0619 | 3.424 | .0076 |
| 4 | 4.434 | .0016* | 8.562 | <.0001* | 5.273 | .0005* | -1.498 | .1684 | 1.610 | .1419 | 1.992 | .0775 |
| 5 | 4.315 | .0019* | 6.119 | .0002* | 4.911 | .0008* | .414 | .6886 | 1.509 | .1655 | 2.217 | .0538 |
| 6 | 2.490 | .0344 | 9.973 | <.0001* | 6.130 | .0002* | -.429 | .6777 | 1.798 | .1057 | 1.345 | .2115 |
| 7 | 4.149 | .0025* | 8.278 | <.0001* | 6.466 | .0001* | .064 | .9505 | 1.836 | .0996 | 2.166 | .0585 |
| 8 | 7.203 | .0001* | 9.450 | <.0001* | 6.361 | .0001* | -1.106 | .2973 | 1.440 | .1837 | 2.821 | .0200 |
| 9 | 3.934 | .0034* | 10.110 | <.0001* | 8.706 | <.0001* | -.352 | .7329 | 2.747 | .0226 | 2.479 | .0350 |
| 10 | 4.641 | .0012* | 9.592 | <.0001* | 6.737 | .0001* | .573 | .5805 | 1.638 | .1358 | 2.035 | .0724 |
| 11 | 1.659 | .1314 | 3.938 | .0034* | 4.755 | .0010* | -1.339 | .2133 | 1.541 | .1576 | 1.713 | .1209 |
| 12 | .573 | .5805 | 3.772 | .0044 | 5.425 | .0004* | -1.299 | .2264 | -.407 | .6932 | .811 | .4384 |
| 13 | 2.128 | .0622 | 2.699 | .0244 | 2.687 | .0249 | .279 | .7863 | .324 | .7530 | .369 | .7240 |

*Note:* Asterisks indicate significant effects between barbell load levels after correction for multiple comparisons (adjusted p – value $p < 0.0038$). Please note that data listed below (t – statistics), corresponds to the topographic images in Figures 2(II) and 3 (II).

**Table 3.** Results of post – hoc paired t – tests between barbell load levels for configuration (C).

| | Post – hoc tests | | | | | | | | | | | |
|---|---|---|---|---|---|---|---|---|---|---|---|---|
| | $HbO_2$ | | | | | | HHb | | | | | |
| | 20% versus 0% | | 40% versus 0% | | 40% versus 20% | | 20% versus 0% | | 40% versus 0% | | 40% versus 20% | |
| Channel | *t* | *p* | *t* | *p* | *t* | *p* | *t* | *p* | *t* | *p* | *t* | *p* |
| 1 | 3.281 | .0095 | 4.102 | .0027 | .2.426 | .0383 | -.7786 | .4562 | .2172 | .8329 | 1.651 | .1331 |
| 2 | 1.174 | .2706 | 1.790 | .1071 | 1.901 | .0897 | -.2980 | .7725 | 1.030 | .3296 | 1.188 | .2652 |
| 3 | .301 | .7703 | 2.242 | .0516 | 2.313 | .0460 | -.2429 | .8135 | 1.083 | .3068 | 1.539 | .1580 |
| 4 | 2.234 | .0524 | 1.857 | .0962 | .240 | .8160 | .6857 | .5102 | .4063 | .6940 | .6327 | .5427 |
| SD | | | | | | | | | | | | |
| 6 | 2.199 | .0555 | 2.716 | .0238 | .973 | .3558 | -.9917 | .3473 | .0085 | .9934 | 1.238 | .2467 |
| 7 | 3.609 | .0057 | 3.505 | .0067 | 1.982 | .0788 | -1.518 | .1632 | .0690 | .9465 | 1.384 | .1996 |
| 8 | 2.448 | .0369 | 4.174 | .0024 | 1.822 | .1017 | -1.609 | .2755 | .0959 | .9257 | 1.186 | .2658 |
| 9 | 3.684 | .0050 | 3.373 | .0082 | 1.153 | .2787 | -3.142 | .0119 | -1.241 | .2460 | 1.413 | .1911 |
| 10 | .860 | .4119 | 1.803 | .1048 | 1.480 | .1729 | -2.938 | .0165 | .0725 | .9438 | 2.132 | .0618 |
| 11 | 1.523 | .1620 | 2.770 | .0217 | 3.273 | .0096 | -.7802 | .4553 | .2543 | .8049 | 1.080 | .3079 |
| 12 | 1.065 | .3146 | 2.655 | .0263 | 3.850 | .0039 | -1.275 | .9013 | 1.751 | .1137 | 1.998 | .0767 |
| 13 | -.507 | .6241 | 2.296 | .0473 | 2.619 | .0278 | .2111 | .8375 | 1.382 | .2003 | 2.813 | .0203 |
| 14 | 1.064 | .3152 | 4.846 | .0009* | 4.350 | .0019* | -1.188 | .2650 | -.2341 | .8201 | .9941 | .3461 |
| 15 | 1.907 | .0889 | 4.946 | .0008* | 4.558 | .0014* | -.6637 | .5235 | -.5846 | .5732 | 1.441 | .1834 |
| 16 | .737 | .4800 | 1.666 | .1301 | 1.339 | .2133 | -1.798 | .1056 | 1.184 | .2667 | 2.004 | .0760 |
| 17 | -1.034 | .3281 | 1.610 | .1418 | 2.838 | .0195 | .9873 | .3493 | 2.289 | .0487 | 1.674 | .1284 |
| 18 | .686 | .5102 | 1.625 | .1386 | 1.503 | .1671 | -2.541 | .0316 | 2.142 | .0608 | 4.026 | .0030 |
| 19 | 1.073 | .3111 | 2.401 | .0398 | 2.642 | .0268 | .1658 | .8720 | 1.991 | .0776 | 4.391 | .0017* |
| 20 | -.534 | .6063 | 1.505 | .1667 | 2.823 | .0199 | .6766 | .5157 | 1.091 | .3034 | .4545 | .6602 |
| 21 | .407 | .6938 | 3.293 | .0093 | 3.647 | .0053 | -.6458 | .5345 | .6470 | .5338 | 3.553 | .0062 |
| 22 | .539 | .6031 | 2.217 | .0539 | 2.479 | .0350 | 1.446 | .1820 | 2.377 | .0414 | 2.064 | .0690 |
| 23 | -.754 | .4701 | 1.596 | .1449 | 3.203 | .0108 | .8617 | .4112 | 2.805 | .0205 | 1.182 | .2672 |

*Note:* Asterisks indicate significant effects between barbell load levels after correction for multiple comparisons (adjusted p – value $p < 0.0023$). Please note that data listed below (t – statistics), correspond to the topographic images in Figures 2 (III) and 3 (III).

**Table 4.** Results of post – hoc paired t – tests between barbell load levels and baseline activity for configuration (A).

| | Post – hoc tests | | | | | | | | | | | |
|---|---|---|---|---|---|---|---|---|---|---|---|---|
| | $HbO_2$ | | | | | | HHb | | | | | |
| | 0% versus BL | | 20% versus BL | | 40% versus BL | | 0% versus BL | | 20% versus BL | | 40% versus BL | |
| Channel | *t* | *p* | *t* | *p* | *t* | *p* | *t* | *p* | *t* | *p* | *t* | *p* |
| 1 | 7.014 | <.0001* | 7.590 | <.0001* | 7.552 | <.0001* | 3.650 | .0053 | 3.112 | 0.125 | 3.298 | .0093 |
| 2 | 3.001 | .0149 | 3.190 | .0110 | 3.443 | .0074 | 2.421 | .0385 | 2.283 | .0484 | 2.887 | .0180 |
| 3 | 4.131 | .0026 | 4.2989 | .0020* | 4.677 | .0012* | .152 | .8822 | .270 | .7932 | 1.540 | .1669 |
| 4 | 3.185 | .0111 | 3.389 | .0080 | 3.573 | .0060 | .088 | .9314 | .521 | .6152 | 1.223 | .2523 |
| 5 | 3.949 | .0034 | 4.528 | .0014* | 5.202 | .0006* | -.957 | .3635 | -.058 | .9551 | 1.126 | .2895 |
| 6 | 4.351 | .0018* | 4.534 | .0014* | 4.848 | .0009* | 1.174 | .2707 | 1.786 | .1077 | 2.271 | .0493 |
| 7 | 3.315 | .0090 | 3.602 | .0057 | 3.956 | .0033 | 1.677 | .1278 | 2.156 | .0594 | 2.925 | .0169 |
| 8 | 4.433 | .0016* | 4.755 | .0010* | 5.068 | .0007* | 1.258 | .2401 | 1.227 | .2509 | 1.628 | .1381 |
| 9 | 3.059 | .0136 | 3.229 | .0103 | 3.435 | .0074 | .631 | .5440 | .268 | .7948 | 1.140 | .2838 |
| 10 | 4.581 | .0013* | 4.900 | .0008* | 5.067 | .0007* | 1.813 | .1033 | 1.493 | .1696 | 1.850 | .0973 |
| 11 | 5.659 | .0003* | 6.270 | <.0001* | 6.958 | .0001* | 1.123 | .2905 | .671 | .5188 | 1.374 | .2026 |
| 12 | 3.185 | .0111 | 3.192 | .0110 | 3.219 | .0105 | 1.702 | .1230 | 1.442 | .1831 | 1.587 | .1470 |
| 13 | 4.140 | .0025 | 4.253 | .0021* | 4.434 | .0016* | 2.775 | .0216 | 2.711 | .0240 | 2.686 | .0250 |
| 14 | 5.493 | .0004* | 5.668 | .0003* | 5.982 | .0002* | 2.316 | .0458 | 2.056 | .0699 | 2.540 | .0317 |
| 15 | 4.609 | .0013* | 4.949 | .0008* | 5.134 | .0006* | 2.458 | .0363 | 2.485 | .0347 | 2.747 | .0226 |
| 16 | 4.944 | .0008* | 5.019 | .0007* | 5.381 | .0004* | 2.056 | .0700 | 1.974 | .0798 | 2.258 | .0504 |
| 17 | 4.960 | .0008* | 5.546 | .0004* | 6.420 | .0001* | .933 | .3751 | 1.199 | .2611 | 1.647 | .1340 |
| 18 | 2.885 | .0180 | 3.009 | .0147 | 3.134 | .0120 | 1.663 | .1308 | 1.738 | .1161 | 2.062 | .0692 |
| 19 | 4.314 | .0020* | 4.609 | .0013* | 5.132 | .0006* | 1.371 | .2035 | 1.619 | .1400 | 2.349 | .0434 |
| 20 | 3.734 | .0047 | 4.237 | .0022* | 4.668 | .0012* | 1.848 | .0977 | 2.188 | .0564 | 3.012 | .0147 |
| 21 | 3.740 | .0046 | 4.052 | .0029 | 4.329 | .0019* | 1.030 | .3300 | .833 | 4.266 | 1.596 | .1449 |
| 22 | 3.064 | .0135 | 3.348 | .0086 | 3.569 | .0060 | 1.405 | .1936 | 1.083 | .3068 | 2.598 | .0288 |

*Note:* Asterisks indicate significant effects between barbell load levels and baseline activity, after correction for multiple comparisons (adjusted p – value $p < 0.0023$). Please note that data listed below (t – statistics), corresponds to the topographic images in Figures 4 (I) and 5 (I).

**Table 5.** Results of post – hoc paired t – tests between barbell load levels and baseline activity for configuration (B).

| | Post – hoc tests | | | | | | | | | | | |
|---|---|---|---|---|---|---|---|---|---|---|---|---|
| | $HbO_2$ | | | | | | HHb | | | | | |
| | 0% versus BL | | 20% versus BL | | 40% versus BL | | 0% versus BL | | 20% versus BL | | 40% versus BL | |
| Channel | *t* | *p* | *t* | *p* | *t* | *p* | *t* | *p* | *t* | *p* | *t* | *p* |
| 1 | 2.103 | .0648 | 2.042 | .0715 | 2.128 | .0623 | -.1186 | .2658 | -1.353 | .2092 | -1.517 | .1636 |
| 2 | 8.881 | <.0001* | 9.246 | <.0001* | 11.577 | <.0001* | 2.900 | .0176 | 2.851 | .0190 | 3.586 | .0059 |
| 3 | 5.320 | .0005* | .5352 | .0005* | 6.050 | .0002* | 1.653 | .1327 | 1.479 | .1732 | 2.110 | .0641 |
| 4 | 7.054 | .0001* | .7149 | .0001* | 8.649 | <.0001* | .977 | .3543 | .365 | .7233 | 2.255 | .0506 |
| 5 | 4.812 | .0010* | 5.063 | .0007* | 5.623 | .0003* | 1.182 | .2675 | 1.186 | .2658 | 1.439 | .1840 |
| 6 | 6.434 | .0001* | 6.474 | .0001* | 8.378 | .0000* | 1.271 | .2357 | .799 | .4449 | 3.198 | .0109 |
| 7 | 10.521 | <.0001* | 11.188 | <.0001* | 11.808 | <.0001* | 2.471 | .0355 | 2.194 | .0559 | 3.002 | .0149 |
| 8 | 5.375 | .0004* | 5.695 | .0003* | 6.798 | .0001* | 1.205 | .2590 | .783 | .4536 | 1.350 | .2099 |
| 9 | 5.187 | .0006* | 5.427 | .0004* | 6.755 | .0001* | 2.679 | .0252 | 2.295 | .0474 | 4.194 | .0023 |
| 10 | 4.200 | .0023* | 4.428 | .0017* | 5.276 | .0005* | .266 | .7965 | .690 | .5077 | 3.114 | .0124 |
| 11 | 12.723 | <.0001* | 12.590 | <.0001* | 13.196 | <.0001* | 1.153 | .2787 | 1.017 | .3356 | 1.259 | .2398 |
| 12 | 4.424 | .0017* | 4.352 | .0018* | 4.618 | .0013* | .762 | .4653 | .307 | .7656 | .492 | .6347 |
| 13 | 2.759 | .0221 | 2.770 | .0217 | 2.780 | .0214 | .886 | .3985 | .830 | .4282 | .792 | .4487 |

*Note:* Asterisks indicate significant effects between barbell load levels and baseline activity, after correction for multiple comparisons (adjusted p – value $p < 0.0038$). Please note that data listed below (t – statistics), corresponds to the topographic images in Figures 4 (II) and 5 (II).

**Table 6.** Results of post – hoc paired t – tests between barbell load levels and baseline activity for configuration (C).

| | Post – hoc tests | | | | | | | | | | | |
|---|---|---|---|---|---|---|---|---|---|---|---|---|
| | $HbO_2$ | | | | | | HHb | | | | | |
| | 0% versus BL | | 20% versus BL | | 40% versus BL | | 0% versus BL | | 20% versus BL | | 40% versus BL | |
| Channel | *t* | *p* | *t* | *p* | *t* | *p* | *t* | *p* | *t* | *p* | *t* | *p* |
| 1 | 2.411 | .0392 | 2.841 | .0194 | 3.256 | .0099 | 1.715 | .1206 | 1.368 | .2046 | 1.668 | .1296 |
| 2 | 1.415 | .1907 | 1.435 | .1850 | 1.618 | .1402 | .498 | .6308 | .412 | .6899 | .697 | .5036 |
| 3 | .646 | .5347 | .635 | .5412 | .951 | .3665 | .278 | .7871 | .169 | .8698 | .434 | .6748 |
| 4 | 1.471 | .1754 | 1.599 | .1444 | .1681 | .1270 | -.943 | .3704 | -.473 | .6473 | -1.004 | .3414 |
| SD | | | | | | | | | | | | |
| 6 | 1.961 | .0815 | 2.205 | .0549 | 2.328 | .0449 | .128 | .9006 | -.060 | .9536 | .108 | .9161 |
| 7 | 1.524 | .1619 | 1.800 | .1054 | 2.117 | .0634 | 2.225 | .0531 | 1.487 | .1711 | 1.878 | .0931 |
| 8 | .995 | .3459 | 1.302 | .2254 | 1.526 | 1.613 | -.314 | .7606 | -.628 | .5457 | -.261 | .8003 |
| 9 | 2.705 | .0242 | 2.961 | .0159 | 3.109 | .0125 | -.331 | .7485 | -.939 | .3720 | -.606 | .5598 |
| 10 | .302 | .7693 | .387 | .7080 | .541 | .6017 | .745 | .4751 | .214 | .8356 | .657 | .5274 |
| 11 | .394 | .7028 | .573 | .5804 | .878 | .4028 | -1.458 | .1787 | -1.672 | 1.289 | -1.310 | .2225 |
| 12 | .178 | .8629 | .279 | .7862 | .509 | .6231 | -.111 | .9140 | -.127 | .9020 | .263 | .7987 |
| 13 | 2.247 | .0512 | 2.260 | .0501 | 2.691 | .0248 | 1.227 | .2511 | 1.069 | .3131 | 1.337 | .2142 |
| 14 | 3.151 | .0117 | 3.445 | .0073 | 3.829 | .0040 | .771 | .4606 | .313 | .7612 | .590 | .5694 |
| 15 | 3.206 | .0107 | 3.424 | .0076 | 3.627 | .0055 | .486 | .6387 | .316 | .7596 | .552 | .5945 |
| 16 | 1.589 | .1466 | 1.614 | .1411 | 1.652 | .1329 | .472 | .6481 | .132 | .8979 | .880 | .4020 |
| 17 | 1.140 | .2839 | 1.072 | .3117 | 1.317 | .2205 | 1.415 | .1908 | 1.625 | .1385 | 1.858 | .0961 |
| 18 | .109 | .9158 | .213 | .8362 | .412 | .6901 | -.049 | .9619 | -.637 | .5398 | .612 | .5554 |
| 19 | 1.226 | .2515 | .1266 | .2373 | 1.549 | .1558 | 2.261 | .0501 | 2.002 | .0763 | 2.523 | .0326 |
| 20 | -1.512 | .1648 | -1.513 | .1645 | -1.168 | .2730 | -.565 | .5861 | -.204 | .8432 | .007 | .9948 |
| 21 | -.162 | .8748 | -.118 | .9089 | .304 | .7682 | .602 | .5619 | .226 | .8265 | .964 | .3604 |
| 22 | .457 | .6582 | .510 | .6222 | .942 | .3710 | -.080 | .9383 | .214 | .8356 | .504 | .6267 |
| 23 | -.498 | .6307 | -.569 | .5831 | -.125 | .9036 | .770 | .4612 | .1057 | .3181 | 1.344 | .2120 |

*Note:* Asterisks indicate significant effects between barbell load levels and baseline activity, after correction for multiple comparisons (adjusted p – values $p < 0.0023$). Please note that data listed below (t – statistics), corresponds to the topographic images in Figures 4(III) and 5 (III).
